# Transcriptional repression is epigenetically marked by H3K9 methylation during SV40 replication

**DOI:** 10.1186/1868-7083-6-21

**Published:** 2014-10-27

**Authors:** Les Kallestad, Kendra Christensen, Emily Woods, Barry Milavetz

**Affiliations:** Department of Basic Sciences, School of Medicine and Health Sciences, University of North Dakota, 501 N Columbia Road, Grand Forks, ND 58203 USA

## Abstract

**Background:**

We have recently shown that T-antigen binding to Site I results in the replication-dependent introduction of H3K9me1 into SV40 chromatin late in infection. Since H3K9me2 and H3K9me3 are also present late in infection, we determined whether their presence was also related to the status of ongoing transcription and replication. Transcription was either inhibited with 5,6-dichloro-1-beta-D-ribofuranosylbenzimidizole (DRB) or stimulated with sodium butyrate and the effects on histone modifications early and late in infection determined. The role of DNA replication was determined by concomitant inhibition of replication with aphidicolin.

**Results:**

We observed that H3K9me2/me3 was specifically introduced when transcription was inhibited during active replication. The introduction of H3K9me2/me3 that occurred when transcription was inhibited was partially blocked when replication was also inhibited. The introduction of H3K9me2/me3 did not require the presence of H3K9me1 since similar results were obtained with the mutant cs1085 whose chromatin contains very little H3K9me1.

**Conclusions:**

Our data suggest that methylation of H3K9 can occur either as a consequence of a specific repressive event such as T-antigen binding to Site I or as a result of a general repression of transcription in the presence of active replication. The results suggest that the nonproductive generation of transcription complexes as occurs following DRB treatment may be recognized by a ‘proof reading’ mechanism, which leads to the specific introduction of H3K9me2 and H3K9me3.

## Background

Five distinct but potentially related elements are thought to contribute to the epigenetic regulation of eukaryotic gene expression: DNA methylation, nucleosome location, histone variation, covalent histone modifications, and interactions by regulatory RNA. Of these elements, the post-translational modification of histones has been of particular interest because of the diversity of the available forms of modification and the number of target amino acids and their physical location within histones. Importantly, there is an abundance of published reports demonstrating the association between certain forms of histone modification and activation or repression of transcription in one or more well characterized biological systems.

In general, it is thought that acetylation of certain lysines in histones is associated with active biological processes like transcription while methylation of lysines or arginines may be associated with either activation or repression of gene transcription. For example, in the nucleosome core, histone H3 acetylation on lysine 9 and 14 (H3K9 and H3K14) have been shown to be associated with transcription along with methylation of lysine 4 (H3K4), while methylation of lysine 9 (H3K9) has been shown to be associated with repression [1, 2].

While the association between the methylation of H3K9 and gene repression has been well established, much less is known about the circumstances that lead to the introduction of methylated H3K9 into chromatin. What has added to this uncertainty is the complexity of methylation, including mono-, di-, and tri-methylation, and the involvement of multiple methylating enzymes [3].

In order to better understand the factors that contribute to epigenetic regulation, we have been investigating the role of histone modifications in the regulation of gene expression during the Simian Virus 40 (SV40) life cycle [4–9]. In a recent publication we confirmed that the mono-methylation of H3K9 in SV40 chromatin was associated with repression of transcription [8]. Specifically, we showed that the introduction of H3K9me1 was a consequence of the repression of early transcription by the product of transcription, T-antigen, binding to a critical regulatory sequence known as Site I [8]. Moreover, importantly, we observed that the introduction of H3K9me1 during repression required DNA replication. Repression occurring prior to the initiation of replication was not associated with the introduction of H3K9me1. Our results demonstrated that replication could serve as an epigenetic switch and that the same biological event could have different epigenetic readouts depending upon whether replication was occurring.

During the course of these studies, we investigated the introduction of all three methylated forms of H3K9 during replication. We noted that unlike the introduction of H3K9me1 which absolutely required replication, the introduction of H3K9me2 and H3K9me3 appeared to occur even when replication was substantially blocked by inhibitor [8]. These results raised two important questions. First, was the introduction of H3K9me2 and H3K9me3 also associated with the repression of transcription in SV40 chromatin, and second, what biological factors contributed to the introduction of these epigenetic marks?

In order to address these questions in SV40 chromatin, we have investigated the effects of a general inhibition or stimulation of transcription on the introduction of H3K9 methylation in the presence or absence of active replication. Transcription was inhibited with 5,6-dichloro-1-beta-D-ribofuranosylbenzimidizole (DRB) [10], a well-characterized inhibitor of RNA Polymerase II (RNAPII) elongation, and stimulated with sodium butyrate, an inhibitor of histone deacetylase [11, 12] known to stimulate gene expression. DNA replication was inhibited with aphidicolin [13]. We show that the introduction of H3K9me2 and H3K9me3 are the result of a general inhibition of transcription at late times in infection but not early times, and that the introduction is partially dependent upon replication but not the prior introduction of H3K9me1.

## Results

### Inhibition of transcription by DRB stimulates the introduction of H3K9me2 and H3K9me3 into SV40 chromatin late in infection but not early in infection

Since we previously observed that the introduction of H3K9me2 and H3K9me3 was not directly related to repression by T-antigen binding to Site I [8], we hypothesized that their incorporation into SV40 chromatin might be the consequence of a more general inhibition of transcription. In order to test this hypothesis we determined the epigenetic consequences of inhibiting transcription with the RNAPII elongation inhibitor 5,6-dichloro-1-beta-D-ribofuranosylbenzimidizole (DRB). We have previously used DRB to investigate the relationship between RNA polymerase II translocation and the acetylation and deacetylation of histones during transcription [7]. For these studies, we chose to investigate SV40 chromatin isolated at 2 hours post-infection when early transcription was occurring but prior to DNA replication, and at 48 hours post-infection when early and late transcription were occurring along with active replication.

**Figure 1:**
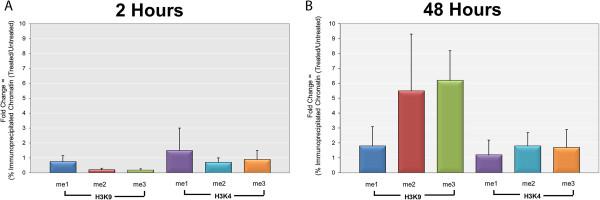
**Inhibition of transcription with 5,6-dichloro-1-beta-D-ribofuranosylbenzimidizole (DRB) stimulates the incorporation of H3K9me2 and H3K9me3 into SV40 chromatin late in infection.** Wild-type SV40 minichromosomes were isolated at 2 hr post-infection with or without incubation with 5,6-dichloro-1-beta-D-ribofuranosylbenzimidizole (DRB) from minus 2 hr until isolation and at 48 hr post-infection with or without incubation with DRB from 24 to 48 hr post-infection. The percentages of intact minichromosomes containing methylated H3K4 and H3K9 were determined by ChIP analyses followed by purification of the intact genomic DNA and PCR amplification with primers recognizing the promoter region. For each form of histone modification, the ratio of the percentage present in the treated minichromosomes compared to the untreated minichromosomes was calculated. The effects of DRB treatment on the introduction of methylated H3K4 and H3K9 from minus 2 hr to isolation at 2 hr post-infection is shown in **(A)**. The corresponding effects of DRB treatment on the introduction of methylated H3K4 and H3K9 from 24 to 28 hr post-infection are shown in **(B)**.

SV40 minichromosomes were isolated and purified from cells infected with wild-type 776 virus following treatment with DRB or no treatment. When minichromosomes were isolated at 2 hours, cells were pretreated with DRB for 2 hours prior to infection, while for minichromosomes isolated at 48 hours post-infection infected cells were treated from 24 to 48 hours. In these experiments, we observed a 112 ± 35 fold increase in the size of the pool of SV40 chromosomes present in glycerol gradient fractions following purification from cells infected for 48 hours compared to 24 hours post-infection. While the increase was somewhat variable we always observed at least a tenfold increase. This increase was reduced 10 ± 6 fold following treatment of the SV40 infected cells with DRB from 24 to 48 hours post-infection. The latter analysis was determined by comparing the increase in the presence and absence of inhibitor and indicates that on average there was about a tenfold reduction in the pool of SV40 minichromosomes following inhibition. As expected, treatment with DRB substantially inhibited the generation of mRNA (data not shown).The effect of DRB on the introduction of methylated H3K4 and H3K9 was determined by subjecting treated and untreated samples of intact SV40 minichromosomes obtained at 2 hours and 48 hours post-infection to ChIP analyses with antibodies that recognize mono-, di-, or tri-methylated H3K4 or H3K9. Intact SV40 minichromosomes were used because they are easily obtained in relatively large amounts and yield the maximum PCR signal compared to fragmented chromatin. Because the SV40 genome was intact, this analysis only yielded information relative to changes in the numbers of minichromosomes carrying a particular modification following treatment. No information was obtained concerning the location of any specific histone modifications. The results of these analyses are graphically shown in Figure 1. The data is displayed as the ratio of the percentage of minichromosomes that contain a modification following treatment divided by the percentage of untreated minichromosomes containing the same modification. If treatment had no effect on the introduction of a particular modification the ratio will be one. Ratios greater than one indicate that treatment results in an increase in the minichromosomes containing the modification while ratios less than one indicate that treatment resulted in a decrease in the presence of a modification.As shown in Figure 1A and B, DRB treatment had no significant effect on the methylation of H3K4 at either 2 hours or 48 hours post-infection. For each form of methylation, the ratio of treated sample to untreated sample was approximately one. In contrast, DRB treatment had a significant effect on the presence of H3K9me2 and H3K9me3 at 48 hours post-infection and little effect at 2 hours post-infection (Figure 1A and B). We observed a 6 ± 4 fold increase for H3K9me2 with a range from 3 to 12 for four independent samples and a 6 ± 2 fold increase for H3K9me3 with a range of 3 to 8 for four independent samples. As expected from our previous published work, DRB treatment had no significant effect on the presence of H3K9me1 at either time point.

### Introduction of H3K9me2 and H3K9me3 following inhibition of transcription is partially dependent upon replication

**Figure 2:**
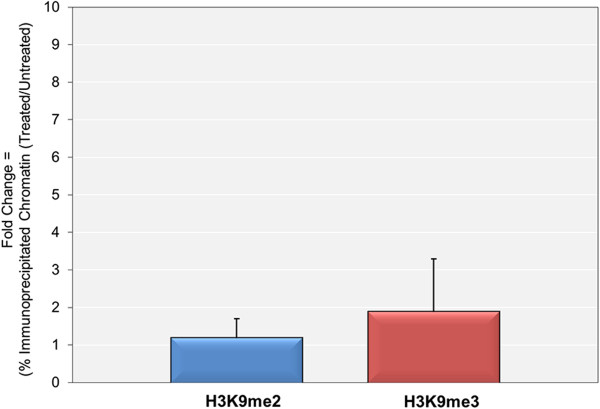
**The 5,6-dichloro-1-beta-D-ribofuranosylbenzimidizole (DRB)-** **stimulated introduction of H3K9me2 and H3K9me3 is partially dependent upon ongoing DNA Replication.** Wild-type SV40 minichromosomes were isolated at 48 hr post-infection with or without treatment with 5,6-dichloro-1-beta-D-ribofuranosylbenzimidizole (DRB) and aphidicolin from 24 to 48 hr post-infection. The treated and untreated intact minichromosomes were subjected to ChIP analyses with antibodies to H3K9me2 and H3K9me3 and the percentages of the minichromosomes containing the modified H3K9s determined by real-time PCR amplification of the bound intact SV40 genomic DNA with primers recognizing the promoter region. The fold increase in the percentages of minichromosomes containing H3K9me2 and H3K9me3 was then calculated.

Since we have previously shown that the introduction of H3K9me2 and H3K9me3 into SV40 chromatin late in infection does not require DNA replication [8], we next tested whether replication played a role in the enhanced introduction of these modifications following DRB inhibition of transcription. SV40 minichromosomes were obtained from infected cells that were untreated or treated from 24 to 48 hours post-infection with a combination of DRB and aphidicolin to inhibit both transcription and replication. The minichromosomes were then subjected to ChIP analyses with antibody to H3K9me2 and H3K9me3 with the results shown in Figure 2. The data is again shown as the ratio between the percentages of input minichromosomes containing the modification of interest in the treated sample compared to the untreated sample. Treatment with aphidicolin and DRB resulted in approximately a 99% reduction in the amount of SV40 minichromsomes obtained at 48 hours post-infection compared to the amount obtained from untreated cells. Following inhibition we observed no increases in the ratio for H3K9me2 (1 ± 0.5) and a small increase in the ratio for H3K9me3 (2 ± 1). These increases were significantly lower than the values obtained when transcription was inhibited while DNA replication was occurring as shown in Figure 1. These results suggest that ongoing replication plays at least a small role in the introduction of H3K9me2 and H3K9me3 at late times in infection.

### Introduction of H3K9me2 and H3K9me3 following inhibition of transcription does not require the presence of H3K9me1

**Figure 3:**
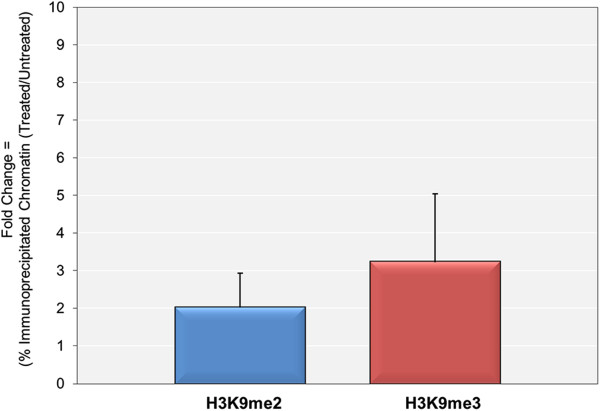
**The 5,6-dichloro-1-beta-D-ribofuranosylbenzimidizole (DRB)-** **stimulated introduction of H3K9me2 and H3K9me3 does not require the presence of H3K9me1.** SV40 minichromosomes from the mutant virus cs1085 were isolated at 48 hr post-infection with or without treatment with 5,6-dichloro-1-beta-D-ribofuranosylbenzimidizole (DRB) from 24 to 48 hr post-infection. Intact minichromosomes were subjected to ChIP analyses with antibody recognizing H3K9me2 and H3K9me3 and the intact SV40 genomic DNA present in the bound fraction quantitated by real-time PCR with primers recognizing the promoter region. The percentages of minichromosomes in the treated and untreated samples were determined and the fold increase in the percentage in the treated samples compared to the untreated calculated.

Since H3K9me1 can be present in as much as 22% of the SV40 minichromosomes present at late times [9], it seemed reasonable that the H3K9me1 containing minichromosomes might serve as substrates for the introduction of H3K9me2 and H3K9me3. To test this possibility we characterized the effect of DRB treatment on the mutant cs1085 SV40 virus. This mutant lacks T-antigen binding Site I and as a consequence fails to down-regulate early transcription. We have previously shown that minichromosomes from this mutant contain very low levels of H3K9me1in contrast to parental wild-type viral chromatin [9]. If the minichromosomes containing H3K9me1 served as a substrate for the introduction of H3K9me2 and H3K9me3, we would expect that inhibition of transcription of cs1085 with DRB would not result in a large increase in the amounts of H3K9me2 and H3K9me3 in the treated minichromosomes compared to the untreated minichromosomes.Cells infected with SV40 cs1085 virus were treated with DRB from 24 to 48 hours post-infection or left untreated and the minichromosomes present in the cells isolated and purified. The minichromosomes were subjected to ChIP analyses with antibodies to H3K9me2 and H3K9me3 with the results shown in Figure 3. Treatment with DRB resulted in significant increases in the amounts of H3K9me2 (3 ± 1 fold) and H3K9me3 (5 ± 3 fold) present in the cs1085 minichromosomes indicating that the presence of H3K9me1 in the minichromosomes was not necessary for the introduction of the higher levels of methylated H3K9.

### Introduction of H3K9me2 and H3K9me3 are associated with SV40 minichromosomes that contain RNAPII

**Figure 4:**
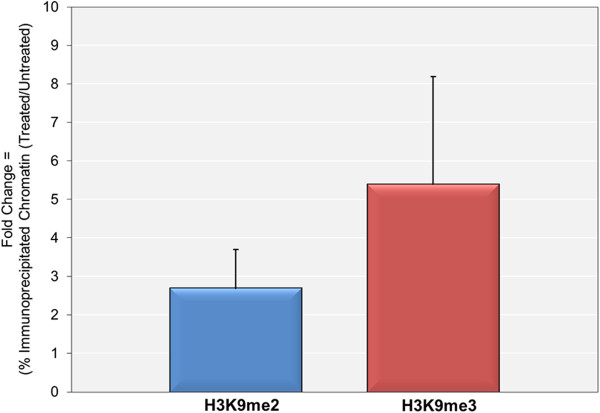
**The 5,6-dichloro-1-beta-D-ribofuranosylbenzimidizole (DRB)-** **stimulated introduction of H3K9me2 and H3K9me3 is partially associated with minichromosomes containing RNAPII.** Wild-type SV40 minichromosomes were isolated from cells at 48 hr post-infection with or without treatment with 5,6-dichloro-1-beta-D-ribofuranosylbenzimidizole (DRB) from 24 to 48 hr post-infection. Intact Minichromosomes were subjected to an ISFIP ChIP analysis in which minichromosomes were first immune selected with antibody to RNAPII. The minichromosomes bound by antibody to RNAPII were sonicated and the soluble chromatin fraction subjected to a second ChIP with antibody to either H3K9me2 or H3K9me3. The percentage of the treated and untreated chromatin containing H3K9me2 and H3K9me3 was determined by real-time PCR using primers that recognize the early region of the genome and the fold increase resulting from treatment calculated.

In order to determine whether H3K9me2 and H3K9me3 were being added to minichromosomes that contain RNAPII following treatment with DRB, we analyzed SV40 minichromosomes that contain RNAPII for the presence of the two methylated forms of H3K9 following treatment with DRB using our ISFIP procedure [4, 5]. In this procedure, SV40 minichromosomes containing RNAPII were immune-selected with antibody to RNAPII bound to protein A agarose in a standard ChIP assay. Following purification of the bound chromatin and prior to elution, the minichromosomes bound to agarose were sonicated to fragment the chromatin into nucleosome-sized pieces and the bound fragments separated from the released fragments. The released fragments were then subjected to a second ChIP with antibodies to H3K9me2 and H3K9me3 to determine whether H3K9me2 and H3K9me3 were present in the minichromosomes containing RNAPII and if so whether the amount changed upon treatment with DRB.As shown in Figure 4 we observed increases in the percentage of the RNAPII containing minichromosomes which also contained H3K9me (2 ± 1 fold) and H3K9me3 (3 ± 2 fold) although not as significant as those observed in Figure 1. These results suggest that minichromosomes containing RNAPII may serve as the substrate for H3K9 methylation.

### Stimulation of transcription with sodium butyrate inhibits the incorporation of H3K9me2 and H3K9me3 into SV40 chromatin late in infection

Since the introduction of H3K9me2 and H3K9me3 appeared to be associated with repression of transcription, we then determined whether stimulation of transcription would have the opposite effect. Sodium butyrate is a well-known inhibitor of many histone deacetylases and for this reason has been used extensively to investigate the relationship between histone acetylation and various biological processes including transcription [11, 12]. We have previously shown that inhibition of histone deacetylases by sodium butyrate results in an increase in transcription of actively transcribed genes as well as an increase in histone acetylation [6, 14]. If increased transcription and/or histone acetylation prevented the introduction of H3K9me2 or H3K9me3, we would expect to see a reduction in the ratio of these two modified forms of H3 in the treated minichromosomes compared to the untreated minichromosomes.

**Figure 5:**
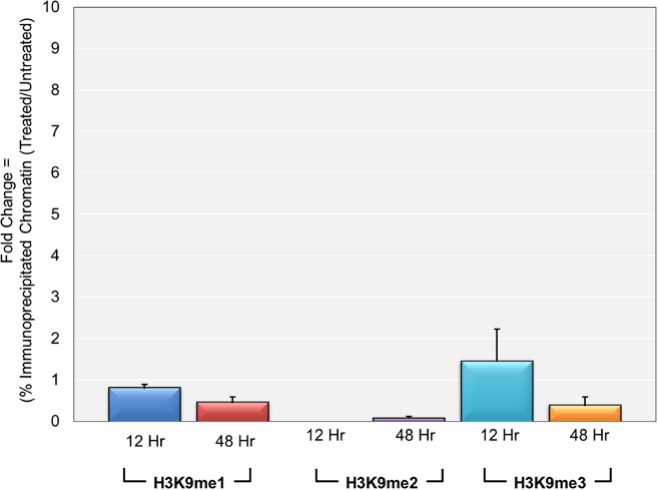
**The introduction of H3K9me2 and H3K9me3 is inhibited by sodium butyrate stimulation of transcription during active replication.** Wild-type SV40 minichromosomes were isolated at 12 hr post-infection with or without treatment from the initiation of infection with sodium butyrate and at 48 hr post-infection with or without treatment with sodium butyrate from 24 to 48 hr post-infection. Intact minichromosomes isolated at 12 hr post-infection were subjected to ChIP analyses with antibodies to methylated H3K9. Intact minichromosomes isolated at 48 h post-infection were subjected to ChIP analyses with antibodies to methylated H3K4 and methylated H3K9. The percentage of treated and untreated minichromosomes containing each form of methylated H3K4 or H3K9 was determined by real-time PCR amplification of the intact SV40 genomic DNA with primers recognizing the promoter region. The fold change resulting from treatment was then calculated from the percentages.

SV40 wild-type infected cells were harvested at 24 hours post-infection, 48 hours post-infection, or 48 hours post-infection following treatment with 50 μM sodium butyrate from 24 to 48 hours post-infection to determine the effects of sodium butyrate during the period of active replication. In a parallel analysis, infected cells were harvested at 12 hours post-infection and 12 hours following a 12-hour treatment with sodium butyrate to determine the effects of sodium butyrate in the absence of replication. The minichromosomes were purified and subjected to ChIP analyses with antibodies to methylated H3K4 and H3K9 with minichromosomes isolated late in infection but only methylated H3K9 when isolated at very early times. The reason for this was our previous observation that relatively little of the SV40 chromatin contained methylated H3K4 at the very early times [9] and because there was no change in the levels of methylated H3K4 following DRB treatment. As shown in Figure 5, we observed ratios close to 1 for H3K9me1 (0.9 ± 0.1) and H3K9me3 (1.5 ± 0.8) in minichromosomes isolated at 12 hours post-infection. We saw very low levels of H3K9me2 in both treated and untreated minichromosomes, which were too variable to quantitate at this time. In contrast, for minichromosomes isolated at 48 hours post-infection, we observed significant inhibition of H3K9 methylation. The ratios were 0.46 ± 0.13 for H3K9me1, 0.08 ± 0.04 for H3K9me2, and 0.39 ± 0.2 for H3K9me3. While H3K4me2 (0.75 ± 0.13) and H3K4me3 (1.26 ± 0.38) did not seem to be affected by sodium butyrate treatment at late times, H3K4me1 (0.47 ± 0.27) appeared to be moderately inhibited (data not show). The decrease in the levels of H3K9me2 and H3K9me3 when transcription was stimulated were consistent with the idea that these modifications were being introduced as a consequence of a general repression of transcription at late times.

### Pools of transcribing SV40 minichromosomes are dynamic during replication

Since DRB had a major effect on transcription and the introduction of H3K9me2 and H3K9me3, we also determined whether it affected the proportion of minichromosomes carrying RNAPII and was, therefore, potentially capable of transcription. We have previously shown that with short-term treatment with DRB there was little effect on the percentage of minichromosomes carrying RNAPII [7]. However, long-term treatment when replication was occurring could be different. In order to better understand the factors contributing to determining the pool size of minichromosomes containing RNAPII during this time frame, we also investigated the effects of aphidicolin and sodium butyrate. If the pool of minichromosomes containing RNAPII was simply related to the overall size of the pool of minichromosomes, we would expect to observe that the percentage containing RNAPII would remain constant despite the overall changes in the size of the pool following the different treatments. For this analysis, we compared the percentage of RNAPII containing minichromosomes at 24 hours post-infection when replication was beginning to 48 hours post-infection when replication was active in the presence or absence of the replication inhibitor aphidicolin, transcription inhibitor DRB, or transcription stimulator sodium butyrate.

**Figure 6:**
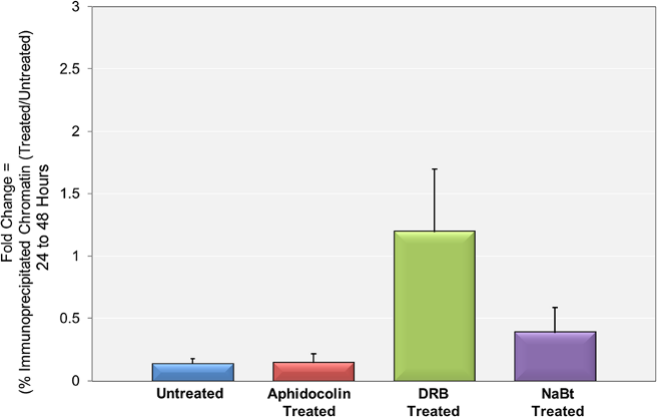
**The pool size of SV40 minichromosomes containing RNAPII is dynamic and dependent upon replication and rate of transcription.** SV40 minichromosomes were isolated from cells infected with wild-type virus at 24 and 48 hr post-infection. Infected cells were either untreated or treated from 24 to 48 hr post-infection with DRB, sodium butyrate (NaBt), or aphidicolin. Intact minichromosomes were subjected to ChIP analyses with antibody to RNAPII and the percentage of untreated and treated minichromosomes containing RNAPII was determined by real-time PCR amplification of the intact SV40 genomic DNA with primers recognizing the promoter region. The fold increase or decrease from 24 to 48 hr post-infection with or without treatment was then calculated from the percentages.

As indicated above, between 24 and 48 hours post-infection we observed a 112 ± 35 fold increase in the size of the minichromosome pool. Following treatment with aphidicolin the pool size at 48 hours post-infection was essentially the same as at 24 hours post-infection indicating substantial inhibition by the drug. Following treatment with DRB we again observed a substantial increase in the pool of minichromosomes although the increase was 10 ± 6 fold less than observed in the absence of the inhibitor. Treatment with sodium butyrate was seen to increase the size of the pool of minichromosomes by 10 ± 4 fold above the level of increase without the added sodium butyrate. The latter two results indicate that the inhibitors had a modest effect on pool sizes when replication was occurring.SV40 minichromosomes from treated or untreated infections were subjected to ChIP analysis with antibody to RNAPII with the results shown in Figure 6. We observed that the ratio of the percentage of minichromosomes containing RNAPII was only 0.14 ± 0.04 comparing minichromosomes isolated at 48 hours post-infection to 24 hours post-infection indicating that only a small fraction of the newly replicated minichromosomes became associated with RNAPII. Following treatment with aphidicolin to block replication the ratio was reduced even further. Compared to untreated controls at 48 hours the ratio was 0.64 ± 0.16. Since there was no increase in the overall pool size this indicated that the actual amount of SV40 chromatin containing RNAPII following inhibition of replication was lower in the treated samples than was present at 24 hours post-infection. Following treatment with DRB from 24 to 48 hours post-infection the ratio of minichromosomes containing RNAPII increased to 1.2 ± 0.5, indicating that the pool size of transcribing minichromosomes was increasing along with replication in the presence of DRB. Finally, we observed that treatment with sodium butyrate during replication also had a profound effect. The ratio of RNAPII containing minichromosomes was 0.39 ± 0.20 following treatment with sodium butyrate from 24 to 48 hours post-infection. This indicated that compared to untreated controls a larger fraction of minichromosomes were capable of transcription.

## Discussion

The introduction of H3K9me2 and H3K9me3 into SV40 chromatin following repression of transcription through the inhibition of RNAPII elongation by the inhibitor DRB is consistent with previous reports showing that the presence of H3K9me2 and H3K9me3 in chromatin is associated with transcriptional repression (reviewed in [15, 16]). H3K9me3, in particular, is now considered a characteristic mark of repression by many laboratories [2].

H3K9me2 and H3K9me3 are typically introduced into chromatin by a member of the SET domain family of histone methyltransferases [16]. The related proteins G9a and GLP are thought to be primarily responsible for H3K9me2 methylation in euchromatin, whereas Suv39H1 is thought to be responsible for H3K9me3 methylation in heterochromatin [17–19]. G9a/GLP also appears to be capable of introducing H3K9me3 into euchromatin [20]. While these proteins contribute to the bulk of the H3K9me2 and H3K9me3 in a cell, there are other methyltranferases that may also play a role since deletion of these enzymes cause a significant but not complete reduction in H3K9 methylation [16].

There are two well-characterized mechanisms for the introduction of H3K9me2 and H3K9me3. H3K9me2 is associated with repression of nuclear hormone receptor regulated transcription (reviewed in [15]). In this model system, the introduction of H3K9me2 is the consequence of the binding of the receptor along with the histone methyltransferase to maintain a repressive environment. Introduction of the ligand results in the selective loss of the methyltranferase and subsequent corresponding loss of H3K9me2 and activation of transcription [15].

The introduction of H3K9me3 into heterochromatin has also been extensively characterized (reviewed in [21]). The introduction of H3K9me3 in heterochromatin is thought to be closely linked with replication and to occur through the targeting of the relevant methyltranferase by associated proteins including CAF1, HP1, and MBD1 in higher eukaryotes [22, 23] and Clr4 and Swi6 in fission yeast [21]. The presence of MBD1 in higher eukaryotes links H3K9me3 to DNA methylation and suggests a possible mechanism for targeting the complex to the appropriate sites (hemi-methylated DNA) following replication.

It seems unlikely that either of these mechanisms is responsible for the introduction of H3K9me2 and H3K9me3 reported here. With respect to the introduction of H3K9me2, SV40 transcription does not appear to be regulated by a nuclear hormone type mechanism as previously described for H3K9me2. Similarly the observed increase in H3K9me2 occurred as a consequence of the inhibition of transcription by DRB not the binding of normal transcription factors. The introduction of H3K9me3 also is unlikely to occur by the same mechanism as described for heterochromatin. Notably, SV40 DNA in chromatin is not known to be methylated because of an absence of DNA methylase substrates in the DNA. Also our observed increase in H3K9me2 and H3K9me3 appeared to be only secondarily a result of DNA replication since the increase was observed on minichromosomes containing RNAPII.

To explain our results we propose the following model. As DNA replication takes place, some of the newly replicated minichromosomes are committed to transcription through the binding of specific and general transcription factors. Concurrently, for reasons as yet unknown, some of the preexisting transcribing minichromosomes stop transcription. These nonfunctioning minichromosomes are recognized as being repressed, and H3K9me2 and H3K9me3 are introduced into their chromatin as a mark of repression. We propose that following replication and the activation for new transcription there is a ‘proofreading mechanism,’ which ensures that the newly initiated minichromosomes are capable of productive transcription. In the event that this is not the case, the minichromosomes are again recognized as repressed, as with the nonfunctioning minichromosomes, and marked by H3K9me2 and H3K9me3. Since treatment with the inhibitor DRB results in a large increase in the number of minichromosomes in which transcription is stopped, presumably at initiation, there is a corresponding increase in the incorporation of H3K9me2 and H3K9me3.

This proofreading mechanism could be specific to SV40 and other similar viruses in which case it could function to ensure that the size of the pool of transcribing minichromosomes is tied to the level of replication and encapsidation. Conversely, this mechanism could be true for higher eukaryotes in general and serve as a way to ensure that when genes are activated for transcription, only those genes correctly activated are allowed to persist in the activated state.

Assuming that the introduction of H3K9me2 and H3K9me3 following the inhibition of elongation by RNAPII reflects a normal biological process for SV40, we believe it is likely to play a role in two aspects of normal SV40 molecular biology. First, the introduction of H3K9me2 and H3K9me3 could play a critical role in regulating the pool size of transcriptionally competent minichromosomes during an infection. We have presented evidence that the pool size is dynamic with new transcribing minichromosomes entering the pool as a result of replication and old minichromosomes leaving the pool. We believe that old minichromosomes leaving the pool of transcribing minichromosomes would be labeled with H3K9me2 and H3K9me3 in order to prevent their reactivation.

Second, H3K9me2 and H3K9me3 could also play a critical role during the initiation of a subsequent infection. We have shown that minichromosomes in virions contain significant amounts of H3K9me2/3 [9]. If the minichromosomes, which contain these modifications, result primarily from the minichromosomes that originally transcribed the late genes following replication, it seems desirable that they be silenced during a new infection when only the transcription of the early genes is required. Allowing transcription of the late genes during the establishment of an infection would seem to be very undesirable for the virus.

Because DRB treatment blocks RNAPII elongation, we believe that it is a way to model the effects of repression of transcription in order to characterize the subsequent changes in epigenetic modifications. However, we recognize that the DRB treatment, as well as the treatment with the other inhibitors themselves, may be responsible for the observed effects on the levels of H3K9me2 and H3K9me3 and may not accurately reflect the epigenetic changes occurring *in vivo*.

## Conclusions

Methylation of H3K9 can occur during active expression either as a consequence of a specific repressive event such as T-antigen binding to Site I or as a result of a general repression of transcription. Moreover, these results suggest that the nonproductive transcription complexes which are generated by DRB treatment may be recognized by a ‘proof reading’ mechanism, which leads to the specific introduction of H3K9me2 and H3K9me3.

## Methods

### Cells and viruses

Wild-type and mutant SV40 minichromosomes were prepared in the monkey kidney BSC-1 cell line (ATCC) using either wild-type 776 virus (from Dr. Daniel Nathans) or cs1085 virus (from Dr. Daniel Nathans).

### Cell culture and infections

BSC-1 cells were maintained and infected as previously described [5–7]. An RNA polymerase II inhibitor, 5,6-dichloro-1-beta-D-ribofuranosylbenzimidizole (DRB), was used at a concentration of 200 μM. For 2-hour infections, DRB was added two hours prior to infection and re-applied at 30 minutes post-infection when the media was replaced by fresh media without virus. For 48-hour infections, DRB was added at 24 hours post-infection. Sodium butyrate was used at a concentration of 250 μM. Sodium butyrate was added at infection and along with fresh media following removal of unbound virus for 12-hour infections and at 24 hours post-infection for 48-hour infections. Replication was inhibited with aphidicolin added at 24 hours post-infection at a final concentration of 6 μM as previously described [8].

### Preparation of SV40 minichromosomes

SV40 minichromosomes were isolated at the indicated times from 2 hours to 48 hours post-infection as described for each of the analyses using our standard purification protocols [5–7] with one minor modification. After transferring the lysed cells to a 15-ml centrifuge tube, an additional one ml of nuclei preparation buffer was used to rinse the flask and was subsequently added to the centrifuge tube in order to maximize the yield of minichromosomes from each infection.

### Chromatin immunoprecipitation

ChIP kits were obtained from Millipore (Temicula, California, USA) and the protocol was followed as previously described [8]. The antibodies used included: H3K4me1 (07 = 436, Millipore (Temicula, California, USA), H3K4me2 (39141, Active Motif Carlsbad, California, USA), H3K4me3 (04 = 745, Millipore Temicula, California, USA), H3K9me1 (ab9045, Abcam, Cambridge Massachusetts USA), H3K9me2 (ab1220, Abcam, Cambridge, Massachusetts, USA), H3K9me3 (ab8898, Abcam, Cambridge, Massachusetts, USA), and RNA PII 905 = 623, Millipore,Temicula, California, USA). All antibodies were ChIP validated by the respective vendors. A total of 100 μl of Protein A agarose, 800 μl chip dilution buffer, and 7.5 μl of each antibody were combined in a protein low-bind tube. The mixture was rotated for 5 hours at 4°C on an end to end rotator in a refrigerator to bind the antibody to Protein A agarose. Following binding of the antibody, the Protein A agarose was centrifuged at 2,000 rpms for 2 minutes and the supernatant discarded. Next, 800 μl of fresh ChIP dilution buffer was added, and 100 μl of the SV40 chromatin to be analyzed was added. The samples containing antibody bound to Protein A agarose and chromatin were incubated with end to end rotation for a further 7 hours at 4°C. The chromatin bound to Protein A agarose was washed according to the manufacturer’s protocol and eluted as previously described [8].

### Immune selection fragmentation immunoprecipitation

ChIPs were performed as previously described [5–7] with the following changes. The amount of input chromatin added was increased to 200 μl and the amount of Protein A agarose was doubled to 200 μl. Following the final wash of the protein A agarose containing antibody and bound chromatin, the agarose was resuspended in 200 μl of buffer and transferred to a clean low-bind Eppendorf centrifuge tube. The suspension was sonicated using a Branson Digital Sonifier for 6 minutes with the amplitude set at 50%. The sonicated samples were centrifuged at 2,000 rpms for 2 minutes to separate the chromatin that remained bound to the agarose from the fragmented chromatin in the supernatant and the supernatant chromatin saved. The chromatin that remained bound to agarose following sonication was removed with lysis buffer according to the ChIP kit’s instructions. The supernatant was then used as the starting material for a subsequent ChIP.

*Preparation of DNA*: Samples were prepared for PCR using an MP Bioscience (Solon, Ohio, USA) Geneclean Spin Kit (#111101-200) with the following modifications. The glassmilk reagent (100 μl) was mixed with 50 μl of sample in a 1.5-ml centrifuge tube. The tube was mixed by repeated inversion at 2 minutes and again at 4 minutes of incubation. Following 5 minutes of room temperature incubation, the samples were centrifuged at 6,000 rpm for 30 seconds in a Micro One (Tomy) to pellet the glass. The supernatant was discarded and 200 μl of the wash buffer was added to the tube. As the wash buffer was being added, the pipette tip was used to break up the pellet by physically rubbing the pellet and vigorously pipetting up and down. The samples were inverted twice and centrifuged at 6,000 rpm for 30 seconds to again pellet the glass. The supernatant was discarded and the pellets were dried in a vacuum for 5 minutes. The glass pellet with bound DNA was resuspended in 25 μl Tris EDTA (TE) buffer.

### PCR amplification

For most of the amplifications, DNA was amplified from the promoter region of the SV40 genome using the primers 5′ TTG CAA AAG CCT CCA AA 3′ and 5′ TGA CCT ACG AAC CTT AAC CGA GGG 3′ in a CFX Connect Real Time System thermal cycler (Bio-Rad, Ipswich, Massachusetts, USA) using ‘SSO Advanced DNA polymerase (Bio-Rad, Ipswich, Massachusetts, USA). In the immune selection fragmentation experiment, DNA was amplified from the early region using the primers 5′TGCTCCCATTCATCAGTTCC3′ and 5′CTGACTTTGGAGGCTTCTGG3′ because the promoter region is extremely sensitive to sonication. Immediately before use, the primers and DNAse free water were added, and 28 μl of the mix was used per sample. 2 μl of the resuspended glass milk in TE buffer was added per sample. Samples were amplified by PCR in triplicate with a melt curve applied afterwards to ensure specific amplification. All sample preparation for PCR was done in either a Nuaire biological safety cabinet Model NU_425-400 or an AirClean 600 PCR Workstation (ISC BioExpress).

### Statement on the use of human subjects

This research did not involve the use of any human subjects, materials, or data.

## Abbreviations

DRB: 5,6-dichloro-1-beta-D-ribofuranosylbenzimidizole
NaBt: sodium butyrate.
